# Engineering T3 and T7 host range to target protein receptors and guide bacterial evolution

**DOI:** 10.1016/j.isci.2025.114299

**Published:** 2025-12-01

**Authors:** Collins Ogari, Kevin Yehl

**Affiliations:** 1 Department of Chemistry and Biochemistry, Miami University, Oxford, OH, USA; 2 Department of Cell, Molecular, and Structural Biology, Miami University, Oxford, OH, USA

**Keywords:** Bact`eriology, Microbial biotechnology, Microbiology

## Abstract

Phage engineering holds significant potential for overcoming the challenges that limit phage therapy. A promising yet underutilized approach is engineering phage host range to target specific receptors and guiding bacterial evolution into a desirable trajectory, referred to as evolutionary steering. This requires balancing binding interactions between native and desired receptors, though it is unclear how balancing binding interactions affects host range and resulting evolutionary trajectories. To provide insights, we surveyed a suite of phage engineering methods to program protein-protein interactions between phage and bacteria and then measured host range expansion and evolutionary trajectories. We engineered T3 and T7, both LPS-targeting phages, to target a proteinaceous nanobody receptor. In addition, we discovered that the capsid plays a role in phage host range and can be a potential target for host range expansion. Together, these studies increase the therapeutic potential for T3 and T7, and more broadly for LPS-targeting phages.

## Highlights

• T3 and T7 were retargeted to bind a nanobody receptor
• Nanobody binding expands T3 and T7 host range
• T7 antigen display on capsid reduces EOP, which is rescued by nanobody expression
• Desired evolutionary steering is guided by target receptor binding and expression

## Graphical abstract

**Figure:**
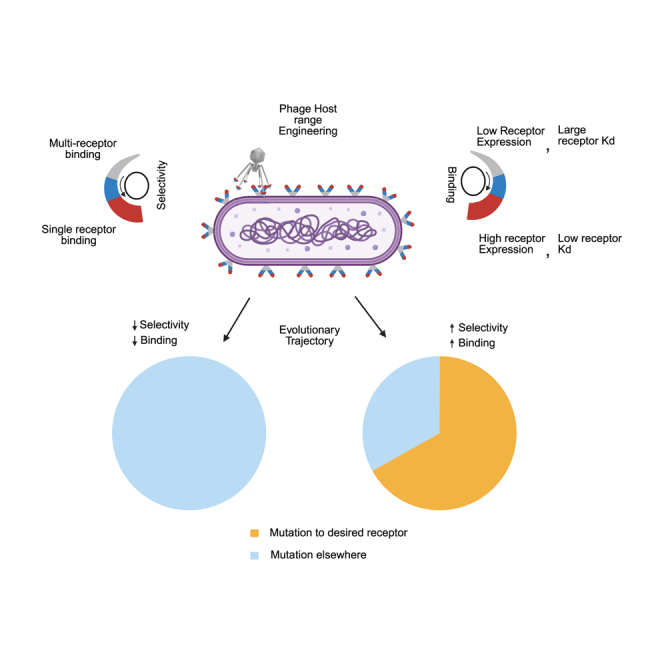

## Introduction

Antibiotic resistance is an urgent public health threat resulting in ∼5 million deaths annually worldwide and is responsible for ∼200,000 deaths in the United States annually.^1^ Worrisome, the incidence of antibiotic-resistant infections is growing due to a complex combination of factors, including over-prescription and misuse of antibiotics, industrial-scale application of antibiotics in livestock, and globalization, increasing the spread of antibiotic-resistant pathogens.

Bacteriophage (phage) therapy, which is the application of viruses to treat infection, offers a promising solution because bacteriophages are insensitive to standard antibiotic resistance mechanisms and can be used to treat multidrug-resistant infections. In addition, phage therapy is targeted, so is specific for a particular bacterial strain, thus preventing dysbiosis. However, selectivity also poses a challenge, as it requires formulating a mixture of bacteriophages into a therapeutic “cocktail” to provide enough coverage to treat the vast diversity of bacterial pathogens, ranging up to hundreds of phages depending on the bacterial species.^2^ This is economically incompatible with the regulatory structure of the United States because each phage requires going through safety testing, which increases costs. Further, if the collection of bacteriophages is extensive, screening is necessary to identify efficacious phages to be formulated into a personalized cocktail for the patient, which poses logistical challenges and also increases costs.^3^^,^^4^ As such, significant efforts are underway to identify bacteriophages with a broad host range that target a wide range of clinical isolates, or to genetically modify phages from current collections that have desirable immunogenic and pharmacokinetic profiles, and extend their host range.^5^^,^^6^

Host range engineering also has potential for retargeting phages to rationally target specific surface molecules on bacteria, such as virulence factors or efflux pumps, which play a significant role in pathogenicity and multidrug resistance, respectively. Virulence factors and efflux pumps are desirable targets because phage resistance can result in loss of pathogenicity or antibiotic resistance, respectively.^7^ However, it is difficult to isolate phages from the environment that bind this class of receptors if they are not readily expressed on the bacterial surface. Engineering phage host range may be the solution for expanding this class of therapy, i.e., rationally guiding bacterial evolution. However, engineering bacteriophages to bind a new receptor requires careful consideration. Phage adsorption requires optimal binding, strong enough to bind permissible hosts, but not too strong, as inhibition can occur from lysed bacterial components.^8^ In addition, balancing native receptor interactions with synthetic receptor binding is important. For example, binding a new receptor may compete with native receptor binding and inhibit phage infectivity.

Herein, we explore this interplay with T3 and T7, both LPS-targeting phages, to bind a nanobody protein fused to an intimin surface receptor, thus demonstrating how phage retargeting can be applied to rationally guide bacterial evolution. This work explores how phage bacterial binding and receptor expression level alter phage infectivity and evolutionary trajectories of bacteria. Together, these studies provide a deeper understanding of the mechanisms governing phage host range, which directly benefits future engineering efforts aimed at developing more effective phage-based therapies and technologies.

## Results

### Screening nanobody and antigen expression level, and multivalent binding interactions

**Figure 1:**
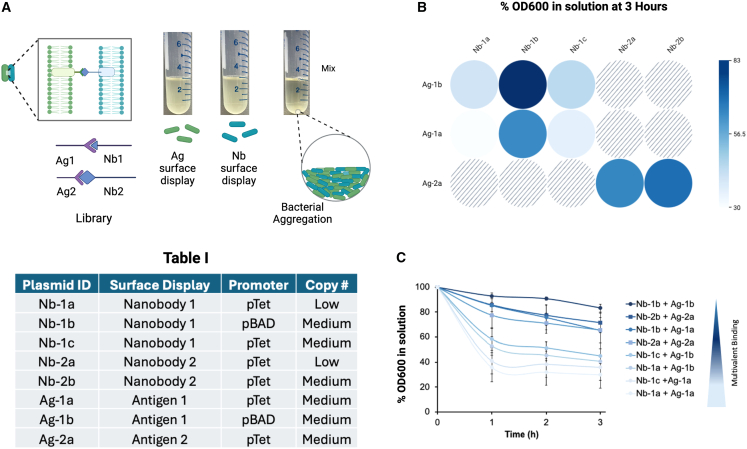
Bacterial surface display platform for screening synthetic receptors with varying affinities (A) Schematic shows the intimin display system and aggregation assay. Table I summarizes the library components. (B) A reactivity array for mixtures of different nanobody antigen binding pairs. Data are shown as a heatmap of %OD_600_ after 3 h. A lower % is indicative of stronger binding. (C) Kinetics for bacterial aggregation and sedimentation. Error bars represent mean ± standard deviation; *n* = 3.

Phage-bacterial binding is dependent upon K_d_ for the bacteriophage’s receptor binding protein and its cognate receptor, valency of interaction, and receptor expression level. To investigate this dynamic, we screened an intimin display library developed by Glass et al. to identify nanobody antigen receptors with varying binding affinities and receptor expression levels. The display library comprises two bacterial strains expressing a nanobody or antigen on the outer bacterial membrane anchored to an intimin receptor (Figure 1A).^9^ Expression of the intimin complex is controlled by arabinose (Ara) or anhydrotetracycline (ATc)- inducible promoters, pBAD or pTet, respectively. Table I in Figure 1 summarizes the library components, which consist of 2 nanobodies (Nb1 and Nb2) and corresponding antigens (Ag1&2), both expressed on low or medium-copy plasmids and from pTet or pBAD promoters (identified as a-c subvariants). The binding efficiency between the nanobody and antigen is measured by inducing the expression of either the nanobody or the antigen, mixing the respective strains together, and tracking bacterial sedimentation over time by monitoring the solution’s optical density. The sedimentation rate is dependent upon the K_d_ of individual nanobody-antigen pairs and expression level (i.e., valency). Complexes that have low K_d_ and high expression of nanobody and antigen will result in faster aggregation and a more rapid decrease in solution’s optical density.

Aggregation was observed in all eight nanobody-antigen pairs immediately after 3 h of mixing (Figure 1B). The data are shown as a percentage of OD_600_ remaining in solution normalized to the OD at *t* = 0 h. Nb-1a & Ag-1a showed the highest binding efficiency, indicated by faster aggregation and a lower % OD, which was surprising since Nb-1a is expressed from a low-copy plasmid (Figure 1A). The subsequent fastest binding was Nb-1c and Ag-1a. Notably, a range of binding efficiencies was observed, including both the rate of sedimentation and steady-state levels (Figure 1C). Next, we engineered these components onto phage and bacteria, so that the binding can be tuned by altering the adhesion surface expression.

### Engineering T3 to display antigen from the tail fiber

Since T3’s native receptor is the inner core LPS,^10^ we initially wanted to test whether binding to a protein receptor would broaden T3’s host range, as binding to a different class of receptor has the potential to inhibit infectivity. T3 was chosen for investigation because it is amenable to engineering, but most importantly, it has a narrow host range. Therefore, host range expansion can clearly be quantified by measuring plaque formation on non-permissive hosts expressing the adhesion complex. Having determined the nanobody-antigen with the strongest multivalent binding interaction, we engineered T3 to display Ag-1a. The antigen was engineered onto T3 because it is much smaller (4AA: EPEA) compared to the nanobody (126AA), so it was expected to have minimal disruption to the tail fiber assembly. However, a nanobody-phage chimera would be a more practical modular scaffold.

**Figure 2:**
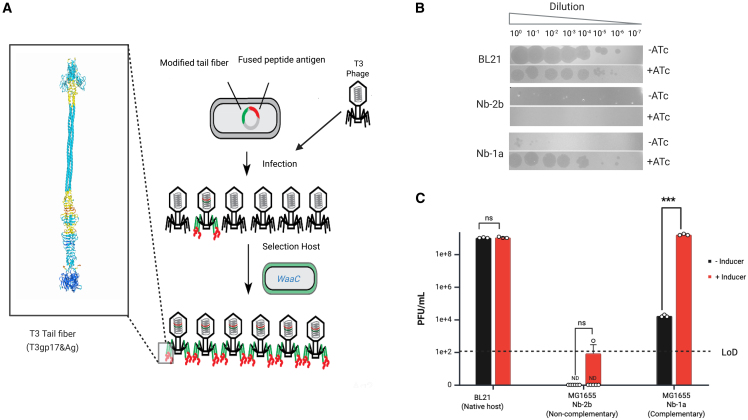
Engineering T3 to display antigen from the tail fiber (A) Schematic shows the recombineering approach to engineer T3 to display Ag-1a from the tail fiber protein gp17. The inset shows an AlphaFold model of the tail fiber complex. (B) Representative image of a plaque assay for recombinant T3-Ag-1a plaqued on BL21 (a permissive host); MG1655 displays a complementary nanobody receptor (Nb-1a); and MG1655 displaying a noncomplementary nanobody receptor (Nb-2b) in the absence and presence of inducer (ATc = 1 ng/mL). (C) Plot quantifies the plaquing results. Black bars show the absence of an inducer, and red bars show + inducer. Error bars represent the mean ± SEM; *n* = 3 (Unpaired *t* test ∗∗∗*p* < 0.001).

Initially, recombineering was performed to engineer T3 to display Ag-1a on the tail fiber C-terminus using homology-directed recombination with a plasmid encoding the engineered tail fiber gene. A schematic summarizing the recombineering strategy is shown in Figure 2A, and the design of the homology arms is shown in Figure S1. Briefly, a mutant tail fiber gene encoding ASRV in the HI loop was cloned into plasmid pT3gp17. The amino acid sequence ASRV was used as a selectable marker to select against wild-type T3 because this mutation is known to extend T3’s host range to infect LPS mutants, BL21Δ*waaC,* and BL21Δ*waaG.*^11^ Next, Ag-1a was cloned onto the C-terminus, including a 2AA spacer (TR) through extension PCR using primers containing overhangs encoding these modifications, which was followed by Gibson assembly into a plasmid vector. The resulting plasmid was transformed into *E. coli* BL21 and recombined into the T3 genome by infecting transformants in the early log phase with T3.

BL21*ΔwaaC* was used to quantify recombination efficiency and select for the engineered variant (Figure 2A, green colored bacteria). To assess host range expansion, plaque-purified phage was amplified in BL21 and used for efficiency of plaquing (EOP) measurements on MG1655 expressing the cognate nanobody. Importantly, T3 does not readily infect MG1655, with an EOP of 4×10^−8^ (Figure S2), so any improvement in plaquing can be attributed to binding between the engineered receptors. Results show an approximate 7-order magnitude improvement in infectivity compared to MG1655 expressing a non-complementary receptor (Figures 2B and 2C). Some leaky expression was observed for uninduced bacteria, as a 2-order of magnitude improvement in plaquing was observed between MG1655 carrying a plasmid encoding Nb-1a in the absence of an inducer, and MG1655 expressing a non-complementary receptor Nb-2b. Importantly, these results show that T3 can be retargeted to bind a protein receptor to enhance infectivity, thus opening the door to targeting other desirable proteinaceous receptors, such as efflux pumps or virulence factors.

### Engineering T7 to display antigen from the tail fiber

Next, T7 was engineered to display Ag-1a because several engineering scaffolds have been developed for the facile genetic modification of T7, which we anticipate will be necessary for future studies of engineering more diverse synthetic receptors onto T7. T7 is also extremely well-characterized genetically and structurally and requires minimal host factors for infectivity, making it an ideal scaffold for phage therapy.

**Figure 3:**
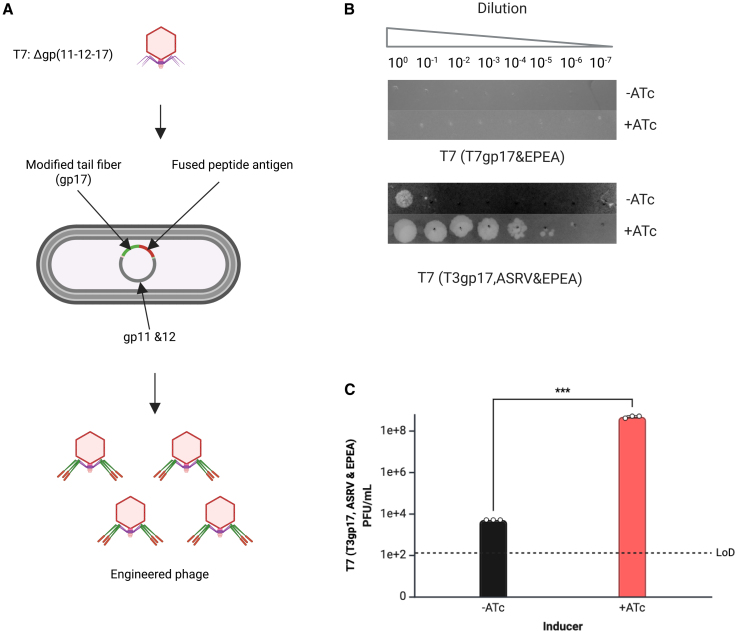
Engineering T7 to display antigen from the tail fiber (A) Schematic shows the complementation-based approach to engineer T7’s tail fiber. (B) Representative images show a plaque assay for T7Δ(11-12-17) complemented with T7gp17_EPEA and plaqued on BL21ΔwaaC harboring a complementation plasmid and an intimin display plasmid encoding for inducible Nb-1c expression (top image). Plaque assay for T7Δ(11-12-17) complemented with T3gp17_ASRV&EPEA tail fiber and plaqued on BL21ΔwaaC harboring a complementation plasmid and an intimin display plasmid encoding for the inducible expression of Nb-1c (bottom image). (C) Bar graph compares plaquing for T7(T3gp17_ASRV&EPEA) on BL21ΔwaaC harboring a complementation plasmid and an intimin display plasmid for inducible (red) vs. non-induced (black) Nb-1c expression. Error bars represent the mean ± SEM; *n* = 3 (Unpaired *t* test ∗∗∗*p* < 0.001).

Initial recombineering efforts to display antigen from T7’s tail fiber protein gp17 were unsuccessful; therefore, we engineered T7 using a complementation system developed by Qimron et al. (Figure 3A).^12^ This engineering strategy bypasses recombination altogether as all phages produced are the engineered variant, so no selection is required.

Briefly, T7Δ(11-12-17) was grown on BW25113 harboring pGEM3RCF, which encodes wild-type T7 gene products 11, 12, and 17. High titers of this lysate were used to infect a day culture of BW25113, which harbored a complementation plasmid encoding the engineered T7 gp17 displaying Ag-1a from the C-terminus and wild-type T7 gene products 11 and 12. Clearing and plaquing were observed on BW25113 harboring the complementation plasmid of the engineered tail fiber (pGEM3RCF_EPEA), indicating correct tail fiber assembly. However, no viable phage was observed when plaquing on BL21Δ*waaC* carrying the complementation plasmid and expressing Nb-1c (Figure 3B, top image). Clearing was also not observed in liquid culture.

A potential explanation could be that the displayed antigen is not accessible for nanobody binding or that binding to an alternative receptor inhibits infectivity. Therefore, we swapped out T7’s tail fiber with that of T3 to display the antigen using the same three-component complementation system, resulting in a T7-T3 hybrid phage. For this phage variant, we did observe successful host range expansion when plaquing on BL21Δ*waaC* carrying plasmids pGEM3RCF_T3gp17-ASRV&EPEA and expressing Nb-1c (Figure 3B, bottom image). A four-order-of-magnitude improvement in plaquing was observed for induced versus uninduced (Figure 3C, bottom image). Together, these results suggest that receptor competition is not the reason for the lack of host range expansion for T7 displaying EPEA antigen.

**Figure 4:**
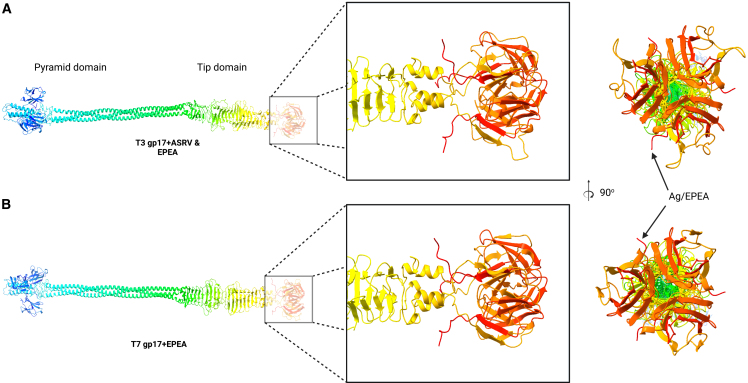
Structure of bacteriophage T3 and T7 tail fibers (A) AlphaFold models of T3 and T7. (B) Engineered tail fiber complexes displaying Ag-1a. Insets show the zoomed-in view of the globular tip domain of the tail fiber complex.

To further investigate host range differences between engineered T3 and T7 displaying Ag-1a from the tail fibers, we compared the AlphaFold structures for both tail fiber complexes. However, no apparent differences were observed (Figure 4A).

### Engineering T7 to display antigen from the capsid

**Figure 5:**
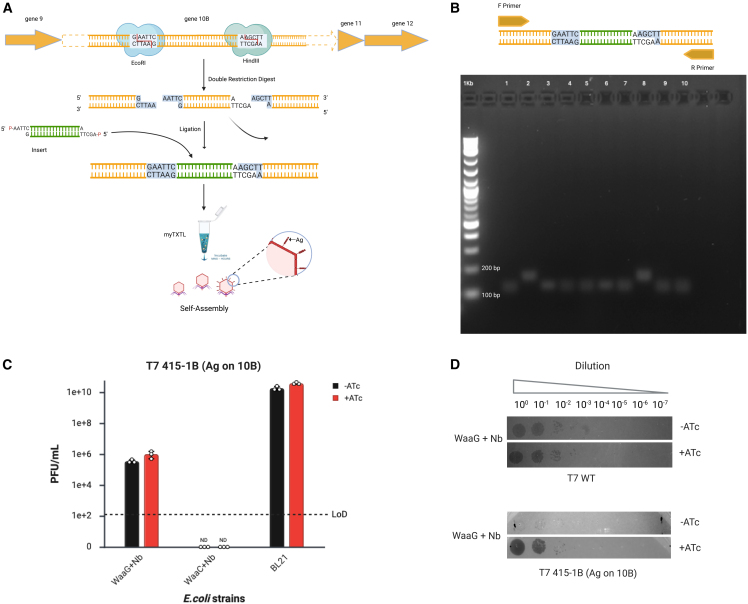
Engineering T7 to display antigen from the capsid (A) Schematic shows steps for engineering T7 select 415-1B to display peptides from the capsid. (B) Schematic shows diagnostic PCR design (top) and corresponding gel electrophoresis results confirming successfully engineered T7. The larger amplicon is indicative of T7 displaying Ag-1a. (C) Bar graph summarizes T7 select 415-1B displaying Ag-1a on capsid plaquing results in *E. coli* strains. Error bars represent the mean ± SEM; *n* = 3. (D) For T7WT and T7 select 415-1B displaying Ag-1a from the capsid.

Next, we engineered T7 to display Ag-1a on the capsid to test whether the capsid can be an engineering target for expanding host range. The capsid is an ideal target, as large proteins can be displayed from the surface and at high valency, thus expanding the receptor repertoire for host range engineering.^13^ Additionally, we would expect that this architecture would not interfere with tail fiber binding to the native receptor. T7 Select 415-1b was used to engineer T7 to display Ag-1a from the capsid protein gp10b. T7 Select system features multiple cloning sites within the capsid protein gene, gp10b, where foreign DNA fragments can selectively be inserted. T7 Select 415-1b can display 415 copies of a peptide up to 50 amino acids in size on capsid protein 10b.^13^ The insert sequence and cloning strategy can be found in Table S1 and Figure 5A, respectively.

Briefly, the insert was ligated into an EcoRI and HindIII digested 415-1b vector and then rebooted using myTXTL cell-free expression system. Successful phage rebooting was indicated by observing a plaquing titer of 2.5×10^4^ PFU/ml on BLT5615 from the reboot reaction. Ten phages were then plaque-purified and screened via diagnostic PCR to distinguish engineered phage variants from empty vector phage (Figure 5B). A 20% successful ligation was observed, indicated by a larger-sized PCR amplicon. Successful ligation was also confirmed through whole-genome sequencing (Figure S3). We believe an 80% empty vector phage is a result of incomplete digestion of the vector.

Next, the capsid-engineered phage was amplified on BL21 and plaqued on BL21Δ*waaG* and BL21ΔwaaC*,* each carrying the Nb-1c intimin display plasmid through inducible expression, to test whether capsid display can be an engineering target for expanding host range. Surprisingly, displaying Ag-1a from the capsid reduced T7 infectivity for BL21*ΔwaaG* absent inducer compared to WT T7 (Figure 5C), which was rescued when BL21*ΔwaaG* was induced to display Nb-1a (Figure 5D). We believe this suggests that T7’s capsid plays a role in phage host range, and engineering it to display Ag-1a interferes with this contribution.

No plaquing was observed for BL21Δ*waaC* for the capsid-engineered phage. We hypothesize that T7 has a weaker binding affinity for BL21Δ*waaC* compared to BL21Δ*waaG,* and that the binding improvement from expressing Ag-1a on the capsid is not enough for infecting BL21Δ*waaC.* Together, these data suggest that the capsid contributes to host range and has the potential to be engineered to broaden phage host range.

### Evolutionary escape

Since the ultimate goal is to guide bacterial evolution, we investigated the evolutionary escape of phage resistant bacterial mutants guided by our retargeted phage. We plaqued the engineered T3 phage displaying Ag-1a from the tail fiber on the MG1655 bacterial host, which contained a plasmid encoding the intimin display system, and selected resistant mutants. Engineered T3 was chosen for evolution studies because it showed the most significant improvement in host range expansion.

**Figure 6:**
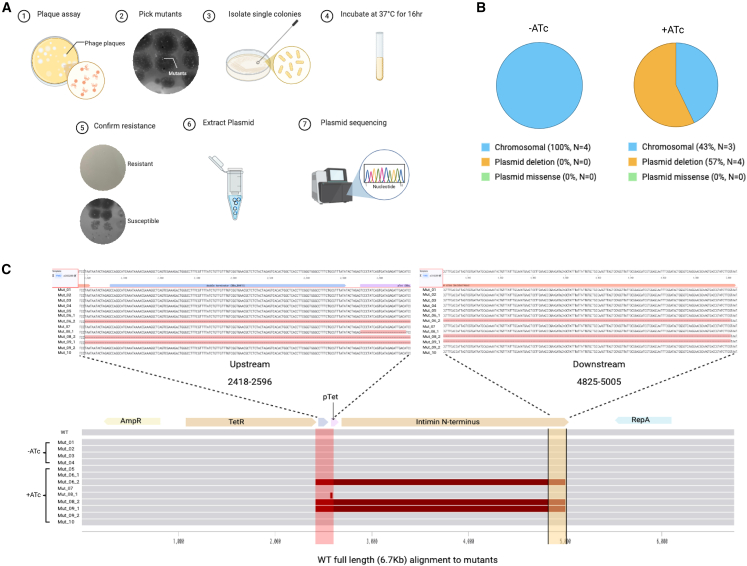
Evolutionary escape (A) Schematic shows steps in experimental work. (B) Pie charts shows the mechanisms of resistance. (C) Sequence alignment image shows the upstream and downstream regions of deletion in the mutants.

To select for phage-resistant bacterial mutants, plaque assays were performed under two conditions: without ATc and with ATc, and both with an increased incubation time to 16 h. Clonal bacterial mutants were isolated, and the intimin plasmids were purified and sequenced by Oxford Nanopore to determine whether the retargeted phage selected for mutations in the receptor gene. Figure 6A summarizes the experimental workflow. Four mutants were isolated as baseline controls in the absence of ATc, and seven mutants were isolated in the presence of ATc. In the presence of ATc, four of the seven bacterial mutants had truncated plasmids, and one of the seven plasmids failed sequencing. The sequencing results for all bacteria, baseline control, and with inducer, are shown in Figure 6C and labeled as Mut_01–10, respectively.

Sequencing of the four baseline mutants (Mut_01–04) isolated without inducer revealed no mutations in the plasmid encoding the intimin-nanobody (Figure 6B). Thus, any phenotypic differences observed in these mutants resulted from chromosomal mutations, likely to the *waa* locus, which encodes for enzymes involved in the assembly of the core lipopolysaccharide. The six mutants isolated in the presence of inducer, where nanobody expression should confer susceptibility, showed diverse genetic changes that prevented phage infection. Three of these mutants (Mut_06, 08 & 09) harbor plasmids having a complete deletion of the intimin-nanobody system (Figure 6C). This deletion eliminates nanobody expression, and therefore, decreases bacterial binding for the engineered phage. Interestingly, no missense mutations were detected (Figure 6B). Also interestingly, mutants Mut_06, 08, and 09 harbored a mixture of plasmids having complete deletion and plasmids with no mutations. The three remaining mutants (Mut_05, 07, and 10) showed no detectable alterations in the plasmid, including the intimin-nanobody system (Figure 6C). This finding suggests that these bacteria harbor mutations elsewhere, potentially in the chromosomal genome, that prevent the engineered phage from infecting the bacteria. Such mutations might alter the expression of T3’s native LPS receptor or nanobody receptor or mutate a vital host gene for phage replication. Whole-genome sequencing would be necessary to pinpoint these adaptive changes.

## Discussion

Phage host range engineering holds significant potential for creating next-generation phage therapeutics that overcome many challenges limiting phage therapy. Engineering the host range of phages is typically achieved through structure-guided engineering or directed evolution of the phage receptor binding proteins (RBPs), which self-assemble into homomeric or heteromeric complexes to form tail fibers or spikes.^14^ For homologous phages, RBPs can readily be swapped to extend host range. For example, T3 and T7 are coliphages that are 89% genetically homologous, and their RBPs are 88% homologous, which can be swapped to extend host range.^15^ This strategy is also generalizable across phage families. For example, Yoichi et al. modified the T2 phage host range by swapping its gene products, gp37 and gp38, located at the tip of T2’s long tail fiber, with PP01’s homologs, which are 91% homologous in the first 150bp and 73% in the last 70bp, thus creating a recombinant T2 phage.^16^ The recombinant T2 phage lost its ability to infect its original host, *E. coli* K12, but gained the capability to infect *E. coli* O157: H7.^17^ T2 wild-type phage infects *E. coli* K12, while PP01 infects *E. coli* O157:H7.

For engineering host range for phages having more distinct relationships, chimeric RBPs are created by combining the recognition domain from the “donor” RBP with the anchoring domain from the “acceptor” RBP. Sometimes a supplementary cognate chaperone is required to ensure proper folding and complex assembly.^18^ It may also be necessary to exchange proteins to which the RBPs are attached to produce a viable phage or swap out other viral components or complexes altogether, such as from the nozzle or baseplate.^19^

Though these approaches extend the phage host range to a native receptor of an RBP, most commonly LPS, they do not rationally target desirable new receptors, such as virulence factors or efflux pumps, which play a significant role in multidrug resistance and pathogenicity.^20^ Engineering phages to bind specific receptors has potential for rationally guiding bacterial evolution as a treatment strategy. However, it is unclear how balancing binding interactions between native and desired receptors affects host range and evolutionary trajectories.

Herein, we studied this interplay. We demonstrate that it is possible to retarget T3 and T7 to bind protein receptors, thereby extending the phage host range. This opens the door to using T3 and T7 to guide bacterial evolution as a novel treatment strategy by targeting efflux pumps and protein virulence factors. We also measured how phage receptor binding and expression alter phage host range. We discovered that T7 displaying antigen from its tail fiber did not extend the T7 host range, where T3 did, thus highlighting nuances in phage engineering, even for well-characterized phages. In addition, T7 phage displaying antigen on the capsid had reduced plaquing efficiency when targeting bacteria not expressing the nanobody receptor. Further studies are needed to assess how the expression of antigen on the capsid affects plaquing, either via structural interference or by changing phage binding orientation.

In conclusion, we showed that the evolutionary trajectories are dependent upon receptor expression and phage binding. For high receptor expression, desired mutations occurred in the plasmid gene encoding the targeted receptor in 57% of the bacterial mutants isolated. The mutations were identified as large deletions in the intimin system, rather than missense mutations, and were generally found as a mixture of plasmids with full deletions versus no mutations. For bacteria having low receptor expression, 100% of the mutations occurred outside the target gene. These findings suggest that retaining binding interactions for the native receptor can provide an evolutionary escape pathway other than mutations to the desired target receptor gene. Therefore, an ideal therapy is one that only binds the target receptor. However, there is a tradeoff if the target receptor is expressed at low levels or expressed transiently.

Quantifiable measures such as these will be crucial for generating a model that correlates K_d_ of individual receptor binding, receptor expression level, and resulting evolutionary trajectories. Such measures and models may help contribute to achieving the grand goal of *de novo* phage design, especially with the rapidly progressing technology of generative protein design. Also, it is interesting from a basic science point of view to study other bacteriophage families. For other phage families, binding to alternative receptors may inhibit host range. Together, these insights will lead to a better-informed selection of therapeutic phage scaffolds for improving the efficacy of phage therapy.

### Limitations of the study

Herein, we developed a model system to measure how phage binding and receptor expression alter infectivity. These results represent initial efforts aimed at creating a quantitative model that links binding strength to host range and resulting evolutionary trajectories. Together, these studies open the door to retargeting T3 and T7 to bind specific receptors on the bacterial surface as a novel treatment modality for evolutionary medicine. Efflux pump-mediated antibiotic-resistant bacterial infections are particularly challenging to treat. Though comprising a small percentage of antibiotic-resistant infections, these infections have high morbidity and mortality. Therefore, they are of high importance to target. Engineering phage host range to target this receptor class as evolutionary guides may be a promising treatment strategy.^21^

Some limitations of this study include the inability to structurally differentiate engineered tail-fibers displaying Ag-1a using AlphaFold. Cryo-EM can be used to study engineered tail fiber complexes to provide the structural insights needed. Another limitation of the study is the lack of in-depth analysis of chromosomal mutations that occur during bacterial evolution when subjected to engineered phages. Mutations discovered on the plasmid do not rule out additional mutations in the chromosome. Whole-genome sequencing can be carried out to better comprehend where the mutations occur in the chromosomes.

## Resource availability

### Lead contact

Further information and requests for resources and reagents should be directed to and will be fulfilled by the lead contact, Kevin Yehl (yehlk@miamioh.edu).

### Materials availability

Further information and requests for resources and reagents should be directed to and will be fulfilled by the lead contact, Kevin Yehl (yehlk@miamioh.edu).

### Data and code availability

• Data: Original raw images, gels, and sequencing data have been deposited at the authors’ institutional repository and are available upon request to the lead contact.
• Code: This study did not generate custom code.
• All other items or information required to reanalyze the data reported in this article are available from the lead contact upon request.

## Acknowledgments

KY would like to acknowledge the support from the 10.13039/100001797PhRMA Foundation. The authors gratefully acknowledge support from the Department of Chemistry and Biochemistry, 10.13039/100006686Miami University.

Schematics were made using BioRender.com.

## Author contributions

C.O. and K.Y. conceived the study; C.O. performed all experiments; C.O. carried out data analysis and interpretation; C.O. and K.Y. wrote the article; supervision and funding, K.Y.

## Declaration of interests

The authors declare no competing financial interests.

## STAR★Methods

### Key resources table

**Table:** 

REAGENT or RESOURCE	SOURCE	IDENTIFIER
**Bacterial and virus strains**		

BL21	This paper	–
BW25113	This paper	–
WaaC	This paper	–
NEB5α	This paper	–
DH5α	This paper	–
WaaG	This paper	–
MG1655	This paper	–
Bacteriophage T7	This paper	T7
Bacteriophage T3	This paper	T3
T7 Select 415-1b	MilliporeSigma	T7

**Chemicals, peptides, and recombinant proteins**		

EcoRI	New England Biolabs	Cat# R3101S
HindIII	New England Biolabs	Cat# R0104S
Polyethylene Glycol (PEG) MW:8000	MilliporeSigma	Cat#25322-68-3
SYBR^TM^ Safe	ThermoFisher	Cat#2687600
Agar	Fisher Bioreagents	Cat#219429
Antibiotics	Goldbio	–
LB Broth	Difco	Cat#1285538

**Critical commercial assays**		

Monarch PCR & DNA Cleanup Kit	New England Biolabs	Cat#T1030
Qiagen Spin Miniprep Kit	Qiagen	Cat#27104
KAPA HiFi HotStart ReadyMix	Roche	Cat#07958935001
myTXTL	–	–

**Oligonucleotides**		

See Table S1	–	–

**Recombinant DNA**		

pDSG290	Glass and Riedel.^9^	GeneBank: MH492375
pDSG289	Glass and Riedel.^9^	GeneBank: MH492377
pDSG398	Glass and Riedel.^9^	GeneBank: MH492453
pDSG360	Glass and Riedel.^9^	GeneBank: MH492448
pDSG310	Glass and Riedel.^9^	GeneBank: MH492381
pDSG375	Glass and Riedel.^9^	GeneBank: MH492440
pDSG312	Glass and Riedel.^9^	GeneBank: MH492380
pDSG419	Glass and Riedel.^9^	GeneBank: MH492413
pT3gp17	This paper	–
pT7gp17	This paper	–
pGEM3RCF	Yosef et al.^12^	–

### Experimental model and study participant details

#### Bacteriophages, strains, and plasmids

Bacteriophages T3 and T7 were obtained from the Yehl lab and maintained on BL21. T7Select®415-1b DNA was obtained from Millipore Sigma, rebooted in myTXTL cell-free extract (Daicel Arbor Biosciences), and maintained on BL21. T7Δ(11-12-17) was obtained from Qimron et al. and maintained on BW25113 harboring a pGEM3RCF plasmid, which encodes for T7 genes *11*, *12*, and *17* for complementation.^12^ BL21Δ*waaC*, BL21Δ*waaG*, BW25113, and MG1655 harboring an intimin display plasmid were used in phage host range studies. The intimin display plasmids were obtained from Glass et al.^9^ Briefly, the intimin system comprises an adhesin construct consisting of a single coding sequence, with a nanobody or antigen as the adhesin, fused to the autotransporter intimin N-terminus from enterohemorrhagic *Escherichia coli*. The display library consists of two nanobodies and corresponding antigens, both expressed on low or medium-copy plasmids. pT3gp17 and pT7gp17 were obtained from the Yehl lab and were used to engineer T3 and T7 through recombineering, respectively. The key resources table lists all the plasmids used in this study.

### Method details

#### Aggregation assay

Bacterial cultures were grown overnight at 37°C while shaking at 250 rpm in 5 mL LB. The next day, 100 μL of overnight culture was diluted in 5 mL of LB +/- 100 ng/mL ATc and grown for 24 hours. Afterward, cultures were briefly vortexed, and 500 μL of culture was combined in a 1.5 mL microcentrifuge tube with another culture at a 1:1 volume ratio and left at room temperature. Samples of 100 μL were collected from the top 25% of the tube and were transferred to a 96-well assay plate to measure optical density at 600 nm (OD_600_) using a BioTek synergy plate reader. Samples were collected and assayed immediately after mixing and every hour for 3 hours.

#### Phage engineering

##### Recombineering

Plasmids pT3gp17 and pT7gp17 were used to engineer T3 and T7 through homologous recombination, respectively. Briefly, mutations to T3’s or T7’s tail fiber gene were introduced through PCR using primers encoding for ASRV in the HI loop of T3 gp17 and Ag-1a at the C-terminus of T3 and T7 gp17, respectively. PCR was performed using KAPA HiFi polymerase (Biosystems) per the manufacturer’s instructions. PCR amplicons of the engineered tail fiber gene and vector backbone were gel purified using a QIAquick gel extraction kit and assembled into a plasmid using Gibson® reaction. Table S1 lists all primers used for cloning. One microliter of the Gibson® reaction was transformed into DH5α and plated onto a selection plate containing the corresponding antibiotic. A single colony was picked from the selection plate and cultured overnight at 37°C, shaking at 250 rpm in 5 ml of LB with the appropriate selection antibiotic. A 100 μL of overnight was diluted in 5 mL of LB lacking antibiotic, which was infected with bacteriophage T3 or T7 at an OD_600_ of ∼0.7 and a MOI of 0.001. The infection was allowed to proceed for no longer than 3 hours to minimize the enrichment of spontaneous phage mutants.

After 2-3 hours, lysates cleared and were treated with 250 μL chloroform (5% final) for 25 minutes to kill any remaining bacteria Phage lysates were then centrifuged at 4,000 rpm for 10 min. The aqueous supernatant was collected, and phage recombinants were acquired by plaquing lysates on *E. coli* BL21Δ*waaC* or *E. coli* BL21Δ*waaC* expressing the corresponding nanobody, which were used as selection hosts for T3 or T7, respectively. Individual plaques were picked and grown on BL21 for amplification, which was stored at 4°C for long-term storage until used for host range studies.

##### Complementation-based approach

T7Δ(11-12-17) was used to engineer T7 to display Ag-1a at the C-terminus of T3 or T7 tail fiber protein gp17. T7Δ(11-12-17) is an engineered T7 with deleted *11*, *12*, and *17* genes. Briefly, T7Δ(11-12-17) was grown on BW25113 harboring pGEM3RCF, which encodes for wild-type T7 gene products 11, 12, and 17. High titers of this lysate were used to infect a day culture of BW25113 harboring a complementation plasmid encoding T7 gene products 11 and 12 and variants of T3 or T7 gp17 displaying Ag-1a at the C-terminus at an OD_600_ of ∼0.7 and MOI of 0.001. pGEM3RCF variant plasmids were cloned using Gibson assembly with corresponding PCR amplicons of T3 or T7 engineered tail fiber genes, respectively. Table S1 lists all primers used for cloning. The co-culture grew until clearing was observed, generally 2-3 hours and no longer than 5 hours. Host range studies were carried out on BL21Δ*waaC*, BL21Δ*waaG*, BW25113, and MG1655 harboring an intimin display plasmid and a pGEM3RCF plasmid encoding for the engineered tail fiber.

##### Genome assembly-based approach

T7Select®415-1b was used to engineer T7 to display Ag-1a on the capsid. T7Select®415-1b is a phage display system that displays 415 copies of a peptide up to 50 amino acids in size on capsid protein 10b.^6^ T7Select®415-1b genome was purified from a 50 mL lysate via phenol-chloroform extraction and ethanol precipitation. Next, the purified genome was double restriction digested using *EcoRI* and *HindIII* restriction enzymes through reactions in 1X rCutSmart^TM^ buffer at a final reaction volume of 50 μl. One microgram of T7Select®415-1b DNA was digested with 20 units of *EcoRI* and *HindIII* at 37°C for 1 hr and heat-denatured. The digested DNA was purified using a Monarch® Spin PCR & DNA Cleanup Kit (NEB). Next, oligonucleotides encoding for Ag-1a were hybridized and ligated into the vector at a molar ratio 5:1 (insert: vector) using 1 μl of T4 ligase in 1X ligase buffer at a total reaction volume 20 μl. The reaction proceeded overnight at 16°C. Three microliters of the heat-denatured ligation reaction were used directly for phage rebooting by mixing with 9 μL of myTXTL and incubating overnight at 29°C. However, no successful phages were produced as determined by plaquing and liquid culture. We postulated that the small insert size resulted in inefficient ligation. Therefore, we designed a larger insert encoding the terminus of gp10b and Ag-1a. We also scaled the reaction to 10 μg digested vector, 3x insert for ligation, and supplemented myTXTL with 3 mM dNTP and 4% PEG final concentration. Together, this resulted in successful phage rebooting.

#### Plaque assay

A fresh bacterial culture of the appropriate host strain was grown overnight at 37°C while shaking at 250 rpm in 5 mL LB plus selection antibiotic and +/- 1 ng/mL inducer. Top agar (0.7% by w/v) was prepared containing antibiotic and +/- ATc and maintained at 60°C in a temperature block. The overnight culture was mixed with the top agar containing the selection antibiotic and +/- inducer at a ratio of 1:10 in a culture tube and immediately poured onto an agar plate also containing the selection antibiotic and +/- inducer. Ten-fold serial dilutions of the phage titer were made in LB in a 96-well plate, and 2 μL of the dilutions were transferred onto the top agar plates using a multichannel pipette, which was left to sit on the bench top for ∼30 min. for the spots to dry. The plates were incubated at 37 °C until plaques were observed, typically after 2-3 hours, depending on the host. Plaques were counted, and the phage titer was calculated using the number of plaques counted, the dilution factor, and the volume of inoculum.

#### Mutant selection and sequencing

A plaque assay was performed at various dilutions, after which the culture plates were incubated for 12-24 hours at 37 °C. Surviving colonies were picked and streaked on fresh culture plates for isolation of single colonies. The isolated colonies were then incubated in liquid culture at 37 °C for 16 hours before performing plaque assays to confirm resistance. Plasmid extraction was done for confirmed resistant mutants using QIAprep® spin Miniprep Kit (250). Whole-plasmid sequencing was done using Oxford Nanopore long-read sequencing technology by Plasmidsaurus.

### Quantification and statistical analysis

#### Statistical analysis

All experiments were performed with a minimum of three replicates, and these values were used to plot mean ± standard deviation. Statistical analysis was performed using BioRender, which uses R (version 4.2.2) for all statistical analyses. Data were analyzed using unpaired t-tests to determine the significance of the results. Unless otherwise stated, results were taken as significantly different by a p-value of <0.001indicated by ∗∗∗.

## Supplemental information

Document S1. Figures S1–S4

Table S1. Excel file listing oligonucleotides used in this study
