# Supplementary material for: Engineering T3 and T7 host range to target protein receptors and guide bacterial evolution
Source: iScience. 2025 Dec 1;29(1):114299. doi: 10.1016/j.isci.2025.114299 (PMC12804162; doi:10.1016/j.isci.2025.114299)
Supplement: Document S1. Figures S1–S4 [file mmc1.pdf]

iScience, Volume *29*

Supplemental information

Engineering T3 and T7 host range to target protein receptors and guide bacterial evolution

Collins Ogari and Kevin Yehl

Supplementary Information

Contents

Fig. S1. Homology arm design for T3 Phage

Fig. S2. T3 plaquing on MG1655 and BL21

Fig. S3. Sequence alignment showing antigen on T7 capsid.

Fig. S4. Sequence alignment of ΔpDSG289 plasmids, Mut_01 – 10.

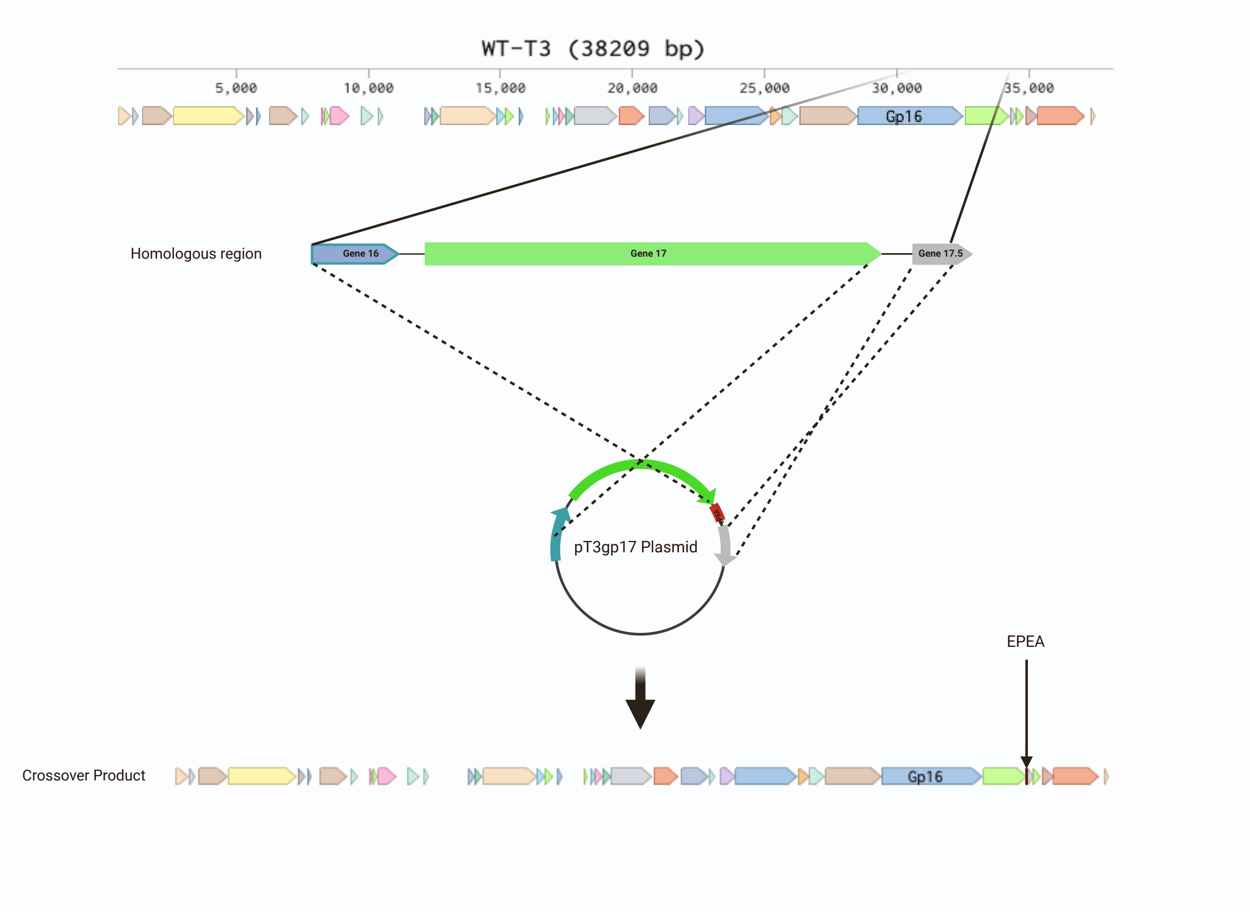

Figure S1: Homology arm design for T3 phage

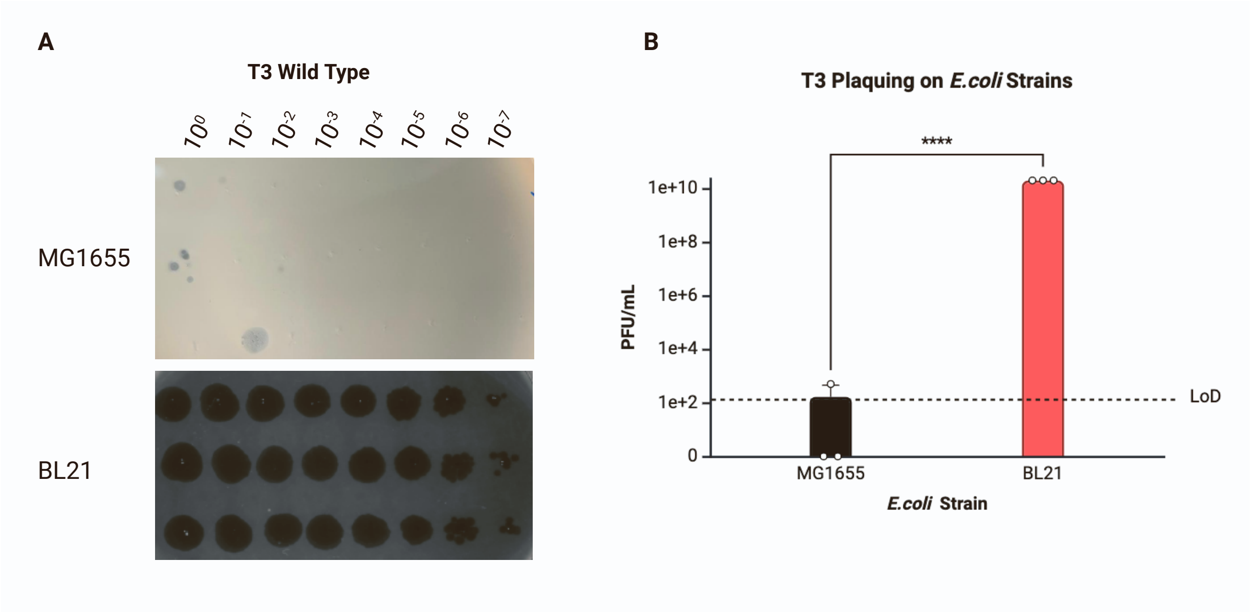

Figure S2: T3 plaquing on MG1655 and BL21. *(A) Plaque assay showing T3 WT plaquing on MG1655 and BL21 and (B) plot summarizing results in (A). (LoD is ~166PFU/ml, Error bars represent the mean ± SEM; n = 3 ***p < 0.001).*

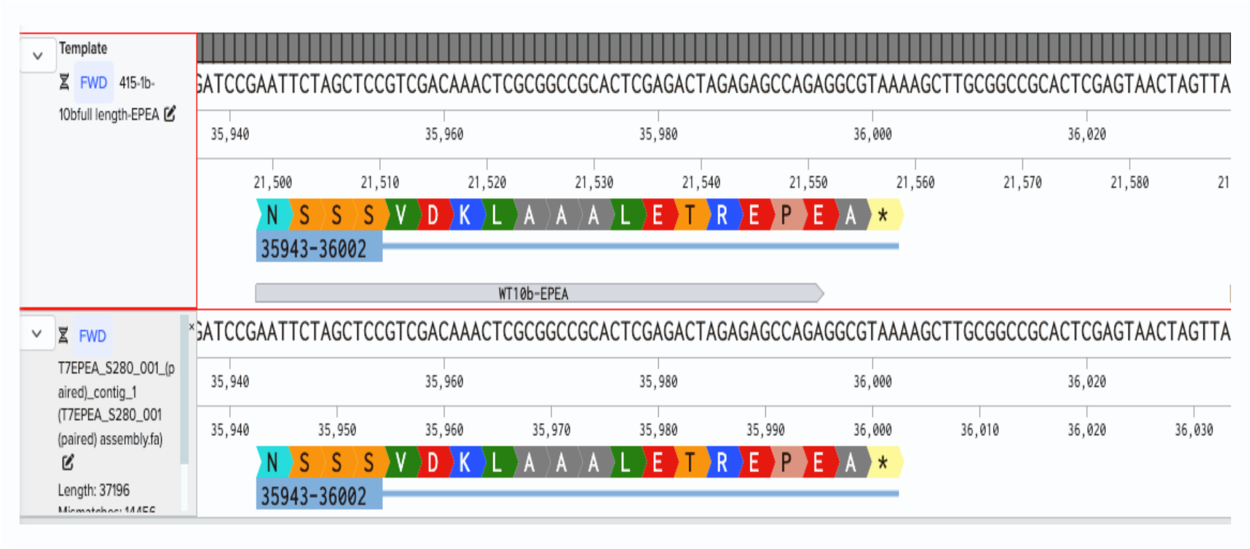

Figure S3: Sequence alignment showing antigen on T7 capsid. *Top sequence is the template sequence.*

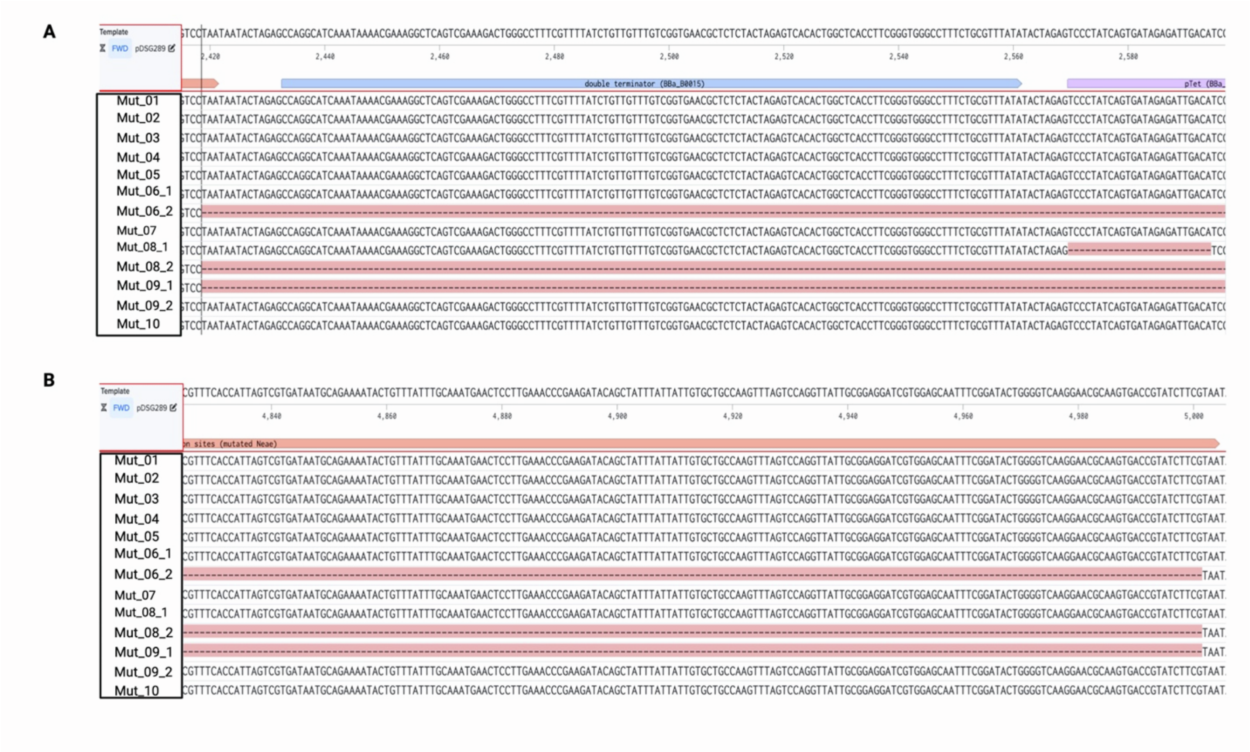

Figure S4: Sequence alignment of ΔpDSG289 plasmids, Mut_01 – 10. *(A) Upstream sequence showing start of deletions in the plasmids and (B) downstream sequence showing end of deletions in plasmids*.
